# Supplementary material for: A systematic review and meta-analysis on pharmacist-led interventions for the management of peptic ulcer disease
Source: PLoS One. 2025 Mar 25;20(3):e0320181. doi: 10.1371/journal.pone.0320181 (PMC11936246; doi:10.1371/journal.pone.0320181)
Supplement: S1 File — (DOCX) [file pone.0320181.s001.docx]

**Supplement 1: Search strategies in databases**

| Participant | Interventions | Comparators | Outcomes |
| --- | --- | --- | --- |
| +18 adults with Peptic Ulcer disease | Pharmacist interventions, medication review, counseling, medication errors, drug interactions, associated factors | Case and control, before and after interventions | Ulcer management and its treatment |
| **Search term** | | | |
| Peptic ulcer, peptic ulcer disease, gastric ulcer, duodenal ulcer, PUD, gastritis, Gastroesophageal reflux disease, Zollinger-Ellison syndrome | Pharmacist interventions, Pharmacist medication review, Pharmacist counseling, medication errors, drug interactions, associated factors, pharmaceutical care | Randomized control trial, case and control |  |
