# Supplementary material for: A systematic review and meta-analysis on pharmacist-led interventions for the management of peptic ulcer disease
Source: PLoS One. 2025 Mar 25;20(3):e0320181. doi: 10.1371/journal.pone.0320181 (PMC11936246; doi:10.1371/journal.pone.0320181)
Supplement: Attachment — Submitted filename: Response to Reviewers.docx [file pone.0320181.s007.docx]

19 November, 2024

Dear Editor and Reviewer,

Greetings of the day,

Thank you for your time and valuable suggestions on our manuscript entitled " A Systematic Review and Meta-Analysis on Pharmacist-Led Interventions for the Management of Peptic Ulcer Disease". We have addressed the reviewer comment and incorporated in the manuscript. We have submitted two separate files of manuscript.

1. In the first file, it is manuscript with track change .

2. The second file is also the revised file, but its clean one

All the revision as requested by the reviewers were addressed and point-to-point answers are given in the response file for each reviewer's questions and comments. We believe that the revised version of the manuscript has significantly improved for the publication.

We humbly request you to kindly inform me, if you need any further corrections from our side in the future.

Thank You.

Corresponding author

**RESPONSE TO JOURNAL REQUIREMENTS 1**

**Journal requirements 1:**

Please ensure that your manuscript meets PLOS ONE's style requirements, including those for file naming. The PLOS ONE style templates can be found at

<https://journals.plos.org/plosone/s/file?id=wjVg/PLOSOne_formatting_sample_main_body.pdf>  and

<https://journals.plos.org/plosone/s/file?id=ba62/PLOSOne_formatting_sample_title_authors_affiliations.pdf>

***Response to journal requirement***

The guidelines were adhered appropriately.

**Journal requirements 2:**

Please include a separate caption for each figure in your manuscript.

***Response to journal requirement***

The procedures were completed in accordance with the guidelines.

**Journal requirements 3:**

As required by our policy on Data Availability, please ensure your manuscript or supplementary information includes the following:

a. A numbered table of all studies identified in the literature search, including those that were excluded from the analyses.

**A separate S2_File named as List of all the studies searched is prepared for this which includes all the list of articles that were searched during our study.**

b. For every excluded study, the table should list the reason(s) for exclusion.

**A separate S3_File named as List of all the excluded studies with explanations is prepared which include all the articles that were excluded for the study with reasons.**

c. If any of the included studies are unpublished, include a link (URL) to the primary source or detailed information about how the content can be accessed.

**NA**

d. A table of all data extracted from the primary research sources for the systematic review and/or meta-analysis. The table must include the following information for each study:
a. Name of data extractors and date of data extraction: **Biswash Sapkota and Bipindra Pandey perform the data extraction from 5/5/2024 to 6/6 2024.**
b. Confirmation that the study was eligible to be included in the review: **Yes**
c. All data extracted from each study for the reported systematic review and/or meta-analysis that would be needed to replicate your analyses.

**Table 1: Characteristics of included studies**

| Name of author and year | Study design and Study site | Study duration | Gender (M/F) | Sample size | Average age (years) | Objectives | Inclusion criteria | reference |
| --- | --- | --- | --- | --- | --- | --- | --- | --- |
| Xin et al., 2018 | Case-control study, Tongde Hospital of Zhejiang province, China | February 1, 2017 - July 31, 2017 | 118/167 | 285 | 59 | To assess whether pharmacological interventions significantly impact the rational use of PPIs and reduce medication costs | Patients admitted to the hospital during the study period, who were at least 18 years old and had current orders for PPIs along with recent laboratory test results | [16] |
| Yailian AL et al., 2022 | Retrospective study, French Hospital | January 1, 2007, to December 31, 2019 | 15011/14683 | 29694 | NR | To evaluate the role of pharmacists in PPI deprescribing, analyze medication-related issues related to PPI prescriptions, examine the associated pharmacist interventions, and identify factors influencing physician acceptance of pharmacist recommendations | Medication order evaluations were conducted over a 12-year period. Within the complete database, pharmacist interventions related to PPIs were extracted. | [17] |
| Chen Q et al, 2022 | Case-control study , General Surgery Department, Fuijan Medical University Hospital, China | June and November,  2018 and between June and November, 2019 | 495/579 | 1074 | 53 | To determine if pharmacist interventions could increase the rate of rational PPI use and reduce the pharmaceutical burden on patients | Patients aged 18 and older admitted to the surgery department with diagnoses related to the esophagus, stomach, duodenum, colon, rectum, small intestine, appendix, hepatobiliary and pancreatic systems, spleen, breast, thyroid, or abdominal hernias | [18] |
| Musuuza JS et al., 2021 | mixed-methods, University of Wisconsin Health Science Hospital, USA | September 2019 to August 2020 | 150/4 | 155 | 71 | To tackle the continuous need for effective strategies that encourage guideline-adherent PPI use and minimize inappropriate use, a deimplementation intervention was created and its feasibility was assessed | Adult patients, who are not in the ICU and have an active PPI prescription, admitted to a medical or surgical unit | [19] |
| Weng A et., 2022 | Case- control study, Telephone intervention, Putian University, Hospital, China | August 2018 to  March 2019 | 80/28 | 108 | 45.5 | To explore the effects of pharmaceutical follow-up on patients with peptic ulcers who have been discharged, examining whether clinical pharmaceutical services can increase patients' understanding of peptic ulcer disease, improve adherence to treatment, and ultimately enhance treatment outcomes and quality of life | Patients aged 18 to 70 years who were discharged from the hospital during the study period with a diagnosis of peptic ulcer verified by electronic gastroscopy | [20] |
| Atkins R et al., 2013 | Retrospective study , Passavant Area Hospital, Jacksonville, Illinois | July 2010 to March 2012 | 432/657 | 1089 | 66.5 | To assess the impact of pharmacy-led training for medical staff on PPI therapy | Retrospective data were gathered from the hospital for patients who were admitted and underwent PPI therapy during the study period | [21] |
| Khalili H et al., 2010 | Exploratory prospective pre- and post-intervention, Imam Khomeini Hospital, Iran | August 1, 2008, to December 1,  2008) and post-intervention (February 1, 2009, to June 1, 2009) | 263/239 | 502 | NR | To evaluate the effect of a clinical pharmacist intervention on promoting and educating about SUP guidelines, and its impact on the appropriate use of AST | Data were gathered from all patients admitted to the infectious disease ward, with a focus on SUP and physician prescribing practices for AST | [22] |
| Buckley et al., 2015 | Retrospective study , ICU and general ward | January 1, 2011, and January 31, 2012 | 568/566 | 1134 | 57 | To evaluate the clinical and economic effects of a new pharmacist-managed stress ulcer prophylaxis program for patients in both the ICU and general ward | All adult patients (aged 18 and older) who were hospitalized and received either an H2 receptor antagonist (H2RA) or a PPI were eligible for inclusion | [23] |
| Tandun et al., 2019 | Retrospective study, Fraser Health Authority (FHA), British Columbia | June 6 to November 12, 2018 | 14/44 | 58 | 80 | To describe the changes and factors influencing a pharmacist-led intervention for deprescribing PPIs in long-term care facilities | A report on residents with active prescriptions for any dosage of a PPI | [24] |

NR= Not reported

d. If data or supporting information were obtained from another source (e.g. correspondence with the author of the original research article), please provide the source of data and dates on which the data/information were obtained by your research group: **NO**
e. If applicable for your analysis, a table showing the completed risk of bias and quality/certainty assessments for each study or outcome.  Please ensure this is provided for each domain or parameter assessed. For example, if you used the Cochrane risk-of-bias tool for randomized trials, provide answers to each of the signalling questions for each study. If you used GRADE to assess certainty of evidence, provide judgements about each of the quality of evidence factor. This should be provided for each outcome:

**In our study we have used modified Newcastle-Ottawa scale for assessing the risk of bias and maintaining the quality of articles included. The table of risk of bais assessment by Newcastle Ottawa scale is as shown below [8].**

**Table 2. Quality scores assessing the risk of bias using a modified Newcastle-Ottawa scale**

| Authors | Representativeness of the sample | Sample  size | Non-respondents | Ascertainment of the exposure | Comparability of subjects in different  outcome groups  (control for  confounding) | Assessment of  the outcome | A statistical test  is appropriate | Total |
| --- | --- | --- | --- | --- | --- | --- | --- | --- |
| Xin et al., 2018 | 0 | 1 | 0 | 1 | 1 | 1 | 1 | 5 |
| Yailian AL et al., 2022 | 1 | 1 | 1 | 1 | 1 | 1 | 1 | 7 |
| Chen Q et al, 2022 | 1 | 1 | 1 | 1 | 1 | 1 | 1 | 7 |
| Musuuza JS et al., 2021 | 1 | 1 | 1 | 1 | 1 | 1 | 1 | 7 |
| Weng A et., 2022 | 0 | 1 | 0 | 1 | 1 | 1 | 1 | 5 |
| Atkins R et al., 2013 | 1 | 1 | 1 | 1 | 1 | 1 | 1 | 7 |
| Khalili H et al., 2010 | 0 | 1 | 0 | 1 | 1 | 1 | 1 | 5 |
| Buckley et al., 2015 | 1 | 1 | 1 | 1 | 1 | 1 | 1 | 7 |
| Tandun et al., 2019 | 0 | 1 | 1 | 1 | 1 | 1 | 1 | 6 |

*(Score: 1 = achieved, 0 = not achieved)*

f. An explanation of how missing data were handled:

**After a thorough review and analysis of the selected articles, if certain information was not adequately provided, it has been indicated as 'Not Reported (NR)'.**

g. This information can be included in the main text, supplementary information, or relevant data repository. Please note that providing these underlying data is a requirement for publication in this journal, and if these data are not provided your manuscript might be rejected.

**Journal requirements 4:**

Please review your reference list to ensure that it is complete and correct. If you have cited papers that have been retracted, please include the rationale for doing so in the manuscript text, or remove these references and replace them with relevant current references. Any changes to the reference list should be mentioned in the rebuttal letter that accompanies your revised manuscript. If you need to cite a retracted article, indicate the article’s retracted status in the References list and also include a citation and full reference for the retraction notice.

***Response to journal requirement***

Thank you for your feedback and requirement. No retracted articles were included in the reference list.

**Additional Editor Comments:**

Thank you for presenting this relevant research work for publication. Although well-written, please pay attention to grammar and spelling errors in the manuscript. For instance, Newcastle on line 110 is misspelled.

***Response to editor comments***

Newcastle-Ottawa Scale (NOS)

**Response to Reviewer 1**

**Comments 1**:

The introduction is long and could be more concise

***Response to reviewer:***

We thank for your valuable suggestions. We agree with reviewer comments and we rewrite the introduction section and make it more concise.

**Comments 2:**

Provide additional details on managing high heterogeneity (e.g., subgroup analysis), which could clarify if the variability across studies affects specific outcomes.

***Response to reviewer:***

Thank you for this question. Regarding high heterogeneity we have employed **I² statistic test** and heterogenicity **Tau² (τ²) among experimental and control group. The sub group analysis was conducted on rational use of medication: *Helicobacter pylori* eradication, intervention types, and age as in table 4. Subgroup analysis revealed that H. pylori eradication yielded a slightly higher effect (0.18) compared to non-eradication (0.16, not significant). Interventions by pharmacists significantly improved rational medication use, with a greater effect observed in direct patient interventions (risk difference = 9.68) compared to lectures (1.49). Age analysis showed higher effects in participants below 50 years (7.12) compared to those above 50 (1.91).** (Table 4).

**Table 4: Subgroup analysis on rational use of medication: *Helicobacter pylori* eradication, intervention types, and age**

| **Outcome/ source** | **No of studies** | **Number of participants** | **Statistical methods** | **Effects size (95% CI)** |
| --- | --- | --- | --- | --- |
| Rational use of medication | 3 [16] [18] [20] | 1467 | Risk difference (M-H, random, 95% CI) | 0.16 (0.04, 0.29) |
| 1. Subgroup: By *H. pylori* eradication |  |  |  |  |
| 1.1. Eradication of *H. pylori* | 1 [20] | 108 | Risk difference (M-H, random, 95% CI) | 0.18 (0.06, 0.30) |
| 1.2 No eradication of *H. pylori* | 2 [16] [18] | 1359 | Risk difference (M-H, random, 95% CI) | 0.16 (-0.02, 0.33) |
| 2. Subgroup : By types of intervention |  |  |  |  |
| 2.1 Lecture to medical staffs and patients by pharmacist | 1 [18] | 1074 | Risk difference (M-H, random, 95% CI) | 1.49 (1.12, 1.99) |
| 2.2 intervention to patients by pharmacist | 2 [16] [20] | 393 | Risk difference (M-H, random, 95% CI) | 9.68 (4.27, 21.93) |
| 3. Subgroup: By age of participants |  |  |  |  |
| 3.1 Age below 50 years old | 1 [20] | 108 | Risk difference (M-H, random, 95% CI) | 7.12 (1.51, 33.56) |
| 3.2 age above 50 years old | 2 [18] [16] |  | Risk difference (M-H, random, 95% CI) | 1.91 (1.46, 2.50) |

Regarding subgroup analysis on duration of therapy it revealed a larger effect in studies within 6 months (SMD = -0.96) and single-center studies (SMD = -0.96). Longer studies (>6 months) and multi-center studies showed moderate effects (SMD = -0.51). Therapy duration was moderately reduced in general wards (SMD = -0.61), but data for surgery wards was not estimated (Table 5).

**Table 5: Subgroup analysis on duration of therapy: Duration of study, Center of study and wards in hospital**

| **Outcome/ source** | **No of studies** | **Number of participants** | **Statistical methods** | **Effects size (95% CI)** |
| --- | --- | --- | --- | --- |
| Duration of therapy | 3 [16] [18] [23] | 2493 | SMD (IV, random, 95% CI) | -0.72 [-1.16, -0.29] |
| 1. Subgroup: By duration of study |  |  |  |  |
| 1.1 Within 6 months | 1 [16] | 285 | SMD (IV, random, 95% CI) | -0.96[-1.20,-0.71] |
| 1.2 More than 6 months | 2 [18] [23] | 2208 | SMD (IV, random, 95% CI) | -0.51[-0.64,-0.39] |
| 2. Subgroup : By center of study |  |  |  |  |
| 2.1 Single Center | 2 [16][23] | 1,359 | SMD (IV, random, 95% CI) | -0.96[-1.20,-0.71] |
| 2.2 Multiple Center | 1 [18] | 1,134 | SMD (IV, random, 95% CI) | -0.51[-0.64,-0.39] |
| 3. Subgroup: By wards in hospital |  |  |  |  |
| 3.1 General and other wards | 2 [16] [23] | 1,419 | SMD (IV, random, 95% CI) | -0.61[-0.72,-0.49] |
| 3.2 Surgery ward only | 1 [18] | 2,493 | SMD (IV, random, 95% CI) | Not estimated |

**Comments 3:**

The interpretation of the results could be made more accessible for broader readership

***Response to reviewer:***

Thank you for your feedback. We will simplify the interpretation of the results by using clearer language, providing brief explanations of statistical terms, and contextualizing the findings for broader understanding.

**Comments 4:**

The authors should standardize terms used throughout the results (e.g., referring to “rational drug use” vs. “appropriate medication use”) for better coherence.

***Response to reviewer:***

Thank you for the feedback. Appropriate medication use was changed to rational use on suitable places.

**Comments 5:**

Some sentences are lengthy, particularly in the Methods and Discussion sections. Breaking these down would improve readability.

***Response to reviewer:***

We thank for your valuable suggestions. We agree with reviewer comments and we have corrected and edited methods and discussion.

**Comments 6:**

Minor grammatical error

***Response to reviewer:***

We thank for your valuable suggestions. We agree with reviewer comments and we have corrected all

Grammatical errors throughout the manuscript.

**Response to Reviewer 2**

**Comments 1:**

Databases for studies published from their inception up to June 2024.Please be specific with the date instead of broad statement ‘’their inception’’.

***Response to reviewer:***

Thank you for your comments. The term inception has been changed to 1904.

**Comments 2:**

Exclusion criteria
#88 Do the authors think that excluding articles not published in English can introduce publication bias?

***Response to reviewer:***

Thank you for your question. Yes, we acknowledge that excluding non-English articles may introduce publication bias; however, this decision was made due to resource limitations and the potential challenges in accurately translating and interpreting non-English studies.

**Comments 3:**

What are the limitations to this study? The authors need to clearly articulate the limitations (strengths and weaknesses of this study).

***Response to reviewer:***

Thank you for your feedback. A key strength of this study is the comprehensive assessment of pharmacist-led interventions in peptic ulcer disease, which provides valuable insights into their impact on rational medication use and patient outcomes. The study followed a rigorous methodology, adhering to PRISMA guidelines, and included a wide range of interventions, enhancing the robustness of the findings. However, there are several limitations. Only English-language studies were included, which may introduce publication bias and limit generalizability. Variability in study design (e.g., case-control, retrospective cohort) likely contributed to heterogeneity, affecting the pooled effect size. Small sample sizes in some studies reduced statistical power, especially in subgroup analyses. Differences in the type, duration, and implementation of pharmacist interventions may have also increased heterogeneity, limiting the consistency of conclusions. Furthermore, the predominance of observational studies, prone to selection bias and confounding, may affect the overall validity of the results.

**Comments 4**
Figure 1: Flow diagram of study selection (Page 5) The authors should please adjust the flow diagram to reflect 9 boxes with clear description (Page MJ, McKenzie JE, Bossuyt PM, Boutron I, Hoffmann TC, Mulrow CD, et al. The PRISMA 2020 statement: an updated guideline for reporting systematic reviews. BMJ 2021;372:n71. doi: 10.1136/bmj.n71)

***Response to reviewer:***

Thank you for your feedback. We have developed PRISMA flow diagram for our study following the given guidelines as follows.

**Identification of studies via databases and registers**

Records removed *before screening*:

Duplicate records removed (n = 165)

Records marked as ineligible by automation tools (n = 0)

Records removed for other reasons (n = 20)

Records identified from:

Databases (n = 5,616)

Registers (n = 0)

**Identification**

Records screened

(n =5431)

Records excluded

(n = 5242)

Reports sought for retrieval

(n =11)

Reports not retrieved

(n = 2)

**Screening**

Reports excluded:

Reason 1 (n = Full text not available)

Reason 2 (n = Not available in English language)

Reason 3 (n = Animal studies)

Reports assessed for eligibility

(n =9)

Studies included in review

(n = 9)

**Included**

**Fig 1. PRISMA flow diagram**
